# Physician's Visiting List for 1880. Lindsay & Blackiston

**Published:** 1880-01

**Authors:** 


*The Physician's Visiting List* for 1880. Publishers: Lindsay & Blackiston, Philadelphia.

This is the twenty-ninth edition of this annual publication, and like its predecessors is a useful engagement book for the dental as well as the medical practitioner. Its contents consist of an almanac, table of signs, Hall's ready method in asphyxia, poisons and their antidotes, the metric system, posological table, and table for calculating the period of utero-gestation ; ending with blank leaves for engagements, visits, memoranda, records, &c.

